# Antimicrobial susceptibility of porcine *Brachyspira hyodysenteriae* and *Brachyspira pilosicoli* isolated in Sweden between 1990 and 2010

**DOI:** 10.1186/1751-0147-54-54

**Published:** 2012-09-21

**Authors:** Märit Pringle, Annica Landén, Helle Ericsson Unnerstad, Benedicta Molander, Björn Bengtsson

**Affiliations:** 1 Department of Animal Health and Antimicrobial Strategies, National Veterinary Institute, Travvägen 20, Uppsala, SE-751 89, Sweden; 2 Swedish Animal Health Service, Box 164, Staffanstorp, SE-245 22, Sweden

**Keywords:** *Brachyspira hyodysenteriae*, Swine dysentery, *Brachyspira pilosicoli*, Porcine intestinal spirochetosis, Antimicrobial susceptibility

## Abstract

**Background:**

The anaerobic spirochetes *Brachyspira hyodysenteriae* and *Brachyspira pilosicoli* cause diarrheal diseases in pigs. Their fastidious nature has hampered standardization of methods for antimicrobial susceptibility testing. For monitoring of antimicrobial susceptibility wild type cutoff values are needed to define where the wild type distribution of MICs ends and no approved cutoffs are available for *Brachyspira* spp. In this study antimicrobial susceptibility data for both species (in total 906 isolates) were compiled and analyzed and wild type cut off values for *B. hyodysenteriae* proposed.

**Methods:**

The MICs of tiamulin, valnemulin, tylosin, tylvalosin, doxycycline and lincomycin were determined by broth dilution in brain heart infusion broth supplemented with 10% fetal calf serum.

**Results:**

The compiled MICs from the broth dilution tests of the *B. hyodysenteriae* type strain, B78^T^ (ATCC® 27164^T^), showed that the method yields reproducible results. In an international perspective the frequencies of isolates with decreased antimicrobial susceptibility were low among both *B. hyodysenteriae* and *B. pilosicoli*. However, in *B. pilosicoli* a constant level of 10-15% isolates with tiamulin MICs >4 μg/ml was detected between 2002 and 2010 and in *B. hyodysenteriae* a gradual increase in tiamulin MICs was seen between 1990 and 2003 although this increase has ceased during the last years. The wild type cutoff values proposed for *B. hyodysenteriae* are: tiamulin >0.25 μg/ml, valnemulin >0.125 μg/ml, tylosin >16 μg/ml, tylvalosin >1 μg/ml, lincomycin >1 μg/ml and doxycycline >0.5 μg/ml.

**Conclusions:**

The broth dilution method used in this study has over the years generated tightly grouped MIC populations for the field isolates and reproducible results for the control strain B78^T^ and is therefore a suitable antimicrobial susceptibility test method for monitoring of *Brachyspira* spp. Here we propose wild type cutoff values for six antimicrobial agents for *B. hyodysenteriae* tested by broth dilution based on MIC distributions and the current knowledge on mechanisms of resistance in this species. There are few studies on antimicrobial resistance mechanisms and MIC distributions in *B. pilosicoli* but to some extent the cutoff values proposed for *B. hyodysenteriae* may be applicable also for monitoring of antimicrobial susceptibility in *B. pilosicoli.*

## Background

The anaerobic spirochete *Brachyspira hyodysenteriae* is the causative agent of swine dysentery [1], a major and serious disease of pigs world-wide. *Brachyspira pilosicoli* causes a milder diarrheal disease in growing pigs, porcine intestinal spirochetosis, which is usually nonfatal but impairs growth rate [2].

Antimicrobial agents such as pleuromutilins, macrolides and lincosamides are important in the control of infections with both *B. hyodysenteriae* and *B. pilosicoli* in pigs. However, development of resistance to these antimicrobial agents is an increasing threat to the treatment options and there are recent reports on multi resistant isolates of *B. hyodysenteriae* from for example Spain and Czech Republic [3,4]. Antimicrobial susceptibility tests of *Brachyspira* spp. are not always performed on a routine basis because of the fastidious nature of these anaerobes and there are no generally approved or recommended standards available. A broth dilution method has been evaluated [5] and is used at the National Veterinary Institute (SVA) in Sweden for monitoring of antimicrobial susceptibility in *Brachyspira* spp. Minimum inhibitory concentration (MIC) quality control ranges for the type strain of *B. hyodysenteriae*, B78^T^ (ATCC® 27164^T^), has been established in an inter-laboratory study for this method [6]. However no criteria for interpretation of the results from susceptibility tests of *Brachyspira* spp. have been officially established.

For monitoring of antimicrobial susceptibility any change in the bacteria that causes decreased susceptibility is important and wild type cutoff values are needed to define where the wild type distribution of MICs ends. Such wild type cutoff values are not intended for guidance of therapy and should not be confused with clinical breakpoints for resistance [7]. The aims of this study were to compile and analyze MICs of six antibiotics for *B. hyodysenteriae* and *B. pilosicoli* isolated between 1990 and 2010 and to propose wild type cutoff values for monitoring of antimicrobial susceptibility of *B. hyodysenteriae*.

## Methods

### Bacterial isolates

**Figure 1:**
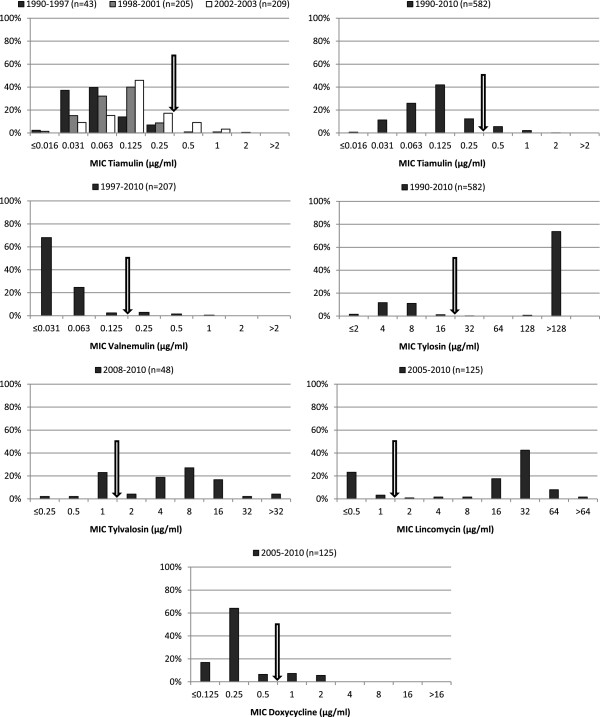
**Antimicrobial susceptibility.** Distribution of MIC of six antimicrobial agents for Swedish field isolates of *B. hyodysenteriae*. Arrows indicate proposed wild type cutoff values.

Swedish field isolates of *B. hyodysenteriae* and *B. pilosicoli*, from clinical submissions to the National Veterinary Institute, Uppsala, between 1990 and 2010 for *B. hyodysenteriae* and between 2002 and 2010 for *B. pilosicoli* were used. Primary isolation and species identification was performed as previously described [8] and the isolates were stored in liquid nitrogen. The antimicrobial agents in the test panels have changed during those years and the number of isolates tested for each antimicrobial agent is presented in Figure 1. For *B. hyodysenteriae* the results from 1990–1999 (72 isolates) have been published previously [5] and for *B. pilosicoli* the results from 2002–2003 (93 isolates) have been published previously [9].

### Antimicrobial susceptibility tests

Thawed isolates were grown on fastidious anaerobe agar supplemented with 10% equine blood (FAA) (National Veterinary Institute, Uppsala, Sweden) and subcultured twice for three days at 37°C prior to susceptibility testing. The purity of all isolates was assessed by phase contrast microscopy. The MICs of tiamulin, valnemulin, tylosin, tylvalosin, doxycycline and lincomycin were determined by broth dilution in VetMIC Brachy panels (National Veterinary Institute, Uppsala, Sweden) as described previously [5]. The medium for the susceptibility tests was brain heart infusion broth (Difco, BD, Sparks, Maryland) supplemented with 10% fetal calf serum. The MIC was read as the lowest concentration of the antimicrobial agent that prevented visible growth. Strain B78^T^ (*B. hyodysenteriae,* ATCC® 27164^T^) was used as control.

## Results and discussion

All tests were performed with the identical broth dilution method and in 96 separate tests the MICs for the control strain were within proposed ranges [6]. For pleuromutilins, this method has been compared with agar dilution [10]. Both methods gave reproducible results, but the broth method on average yielded a one two-fold dilution lower MICs.

**Figure 2:**
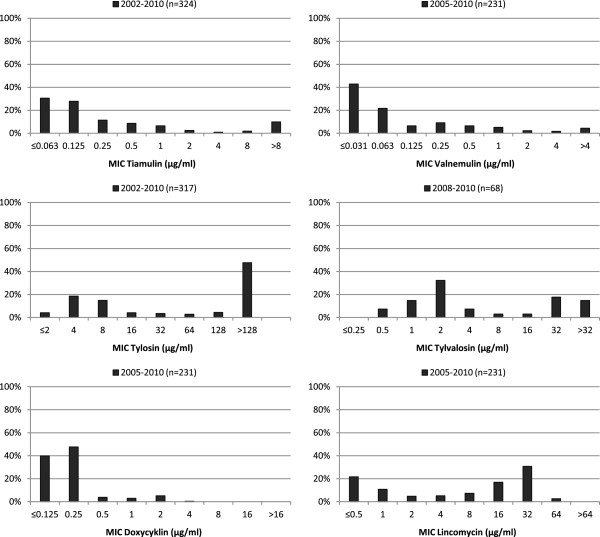
**Antimicrobial susceptibility.** Distribution of MIC of six antimicrobial agents for Swedish field isolates of *B. pilosicoli*.

When viewed from an international perspective the frequencies of isolates with decreased susceptibility to the tested antimicrobial agents are low among Swedish *B. hyodysenteriae* and *B. pilosicoli* (Figure 1 and 2). Compared to Spain and the Czech Republic where even multi resistant isolates are found the situation in Sweden is very favorable [3,4]. Additionally, the frequencies of isolates with decreased susceptibility have been stable over time for most of the antimicrobial agents. Nonetheless, during the period 1990–2003 a gradual increase of isolates with elevated tiamulin MICs was detected for *B. hyodysenteriae* (Figure 1). However, this increase has ceased and the results from 2004–2010 are comparable to the results from 2002–2003.

Tiamulin was introduced on the Swedish market in 1988 [11] and during the 1990s the use increased markedly [12]. During those years the major indication for usage of tiamulin was swine dysentery. A control program against swine dysentery was launched in Sweden by the Swedish Animal Health Service year 2000 and since then the number of *B. hyodysenteriae* positive samples at SVA has been decreasing [13,14]. This coincides with a decrease in use of pleuromutilins which currently has returned to the levels of the first years on the Swedish market for tiamulin [12]. In the control program nucleus and multiplying herds are certified as free from swine dysentery by going through a sampling and observation period of six months. If *B. hyodysenteriae* is detected an eradication program is conducted. When a herd is certified as free yearly negative samplings of pigs susceptible to swine dysentery (20–40 kg body weight) are required to retain the certification [15]. Based on remitted samples, at present less than 1% of the herds in Sweden are diagnosed with swine dysentery and if the number of unrecorded cases is not too large, which the low usage of pleuromutilins contradicts, an eradication of the disease from the whole country may be possible.

The ceased increase in elevated tiamulin MICs may be explained by the strategy within the control program. During eradication of *B. hyodysenteriae* from a herd a plan is made how to clean the stables and treat the pigs with tiamulin allowing only treated pigs to be moved to clean areas until the entire herd is free. This is probably the most favorable way of antibiotic use to avoid development of resistance, because even if a substantial amount of tiamulin is used during sanitation of a herd it is a single event for a limited period. In contrast, continuous treatment with antibiotics in herds with swine dysentery over extended periods of time is the probable explanation to the situation of resistance reported in Spain and the Czech Republic.

There was no change in tiamulin MIC over time for *B. pilosicoli* (data not shown) and in contrast to *B. hyodysenteriae*, a constant level of approximately 10-15% of clinically tiamulin resistant *B. pilosicoli* (MIC 8–32 μg/ml) isolates was detected during the years 2002–2010. In this study no *B. pilosicoli* isolates from the 1990s were included but in a previous study of *B. pilosicoli* (n = 41) from 1990–2001, the tiamulin MIC was 8–32 μg/ml for 9% of the isolates. No mechanism has yet been described for pleuromutilin resistance in *B. pilosicoli*.

Decreased susceptibility to tylosin in *B. hyodysenteriae* and *B. pilosicoli* is caused by mutations in the 23S ribosomal RNA gene which inhibit the binding of the drug to its target [16,17]. One single mutation changes the tylosin MIC from 4–8 μg/ml to >256 μg/ml which generates a clear bimodal distribution (Figure 1). This mutation can be selected for within a few passages *in vitro* and consequently a high proportion of such isolates is found in the field. We have previously suggested that cross-resistance occurs between tylosin and tylvalosin in *B. hyodysenteriae *[18]. The MIC population of the isolates tested for tylvalosin susceptibility in this study (n = 48) showed a tendency to a trimodal distribution with the peaks at 1, 8 and >32 μg/ml. In Table 1 the relationship between the tylvalosin, tylosin and lincomycin MICs is presented. All 11 isolates that had tylvalosin MIC 1 μg/ml were tylosin susceptible (MIC 4–8 μg/ml) and all 35 isolates with tylvalosin MIC >1 μg/ml had tylosin MICs >64 μg/ml. The lincomycin MICs followed the results for the macrolides. However two isolates with high tylosin MICs deviated with an unusual susceptibility pattern, having high lincomycin MICs but lower tylvalosin MICs than the wild type population. Possibly, different combinations of mutations in the 23S rRNA gene could explain both the highest tylvalosin MICs and the pattern for the two deviating isolates but this needs to be studied further.

**Table 1:** **Comparison between tylosin, lincomycin and tylvalosin MICs for the 48 isolates of *Brachyspira hyodysenteriae *susceptibility tested against tylvalosin**

**No. of isolates**	**MIC (μg/ml)**		
	**Tylosin**	**Lincomycin**	**Tylvalosin**
35	>64	16->64	2->32
11	4-8	0.5-1	1
2	>128	32	0.25-0.5

For *B. pilosicoli* all isolates with tylvalosin MIC >16 μg/ml also had tylosin MICs >64 μg/ml but isolates with lower tylvalosin MICs did not follow the pattern seen for *B. hyodysenteriae*. This suggests different mechanisms or different combinations of mechanisms for the decreased tylvalosin susceptibility between the two species.

Decreased susceptibility to doxycycline has been associated with a mutation in the 16S rRNA gene position 1058 (*E. coli* numbering) for *Brachyspira intermedia* isolated from poultry and for *B. hyodysenteriae*[19,20]. The doxycycline MIC for isolates with a G1058C mutation is 1–2 μg/ml whereas the MIC for the wild type population is 0.063-0.5 μg/ml. Among the *B. hyodysenteriae* and *B. pilosicoli* isolates in this study 13% and 9% respectively had a doxycycline MIC >0.5 μg/ml.

In *Brachyspira* spp. decreased susceptibility to tiamulin develops in a stepwise manner for which the genetic background is only described in part. Several mutations altering the binding site at the ribosome have been found [3,21]. The mutations are present in different combinations causing different levels of increased tiamulin MIC and there are isolates with high tiamulin MICs in which none of the known changes can be found. This mix of clones with different susceptibility causes a trailing endpoint in MIC distribution diagrams and difficulties to define a wild type cutoff value to separate the wild type from isolates with acquired decreased susceptibility. For valnemulin the MICs follow the tiamulin MICs in most cases but are generally a few dilution steps lower. In Figure 1 the MICs of tiamulin for *B. hyodysenteriae* are shown in two diagrams, one with all 582 isolates together and one with three subpopulations of in total 457 isolates (1990–1997, 1998–2001 and 2002–2003). For the first subpopulation, 1990–1997, the peak of the susceptible population is at an MIC of 0.031-0.063 μg/ml but if the population is not divided the susceptible peak appears to be at 0.125 μg/ml. Because the mechanisms of tiamulin resistance are only partly known this masked decreased susceptibility can only be detected if monitoring begins in time when a sufficient portion of a population under selection pressure are still of wild type.

For monitoring of resistance it is more important to detect the low-level resistance (or decreased susceptibility) than to find the isolates with the highest MICs. The low-level resistance could be the first step towards higher MICs and hence all the more important to control. To monitor a gradual decrease in susceptibility such as for tiamulin in *Brachyspira* spp. a wild type cutoff value close to the edge of the susceptible wild type distribution is needed, which would be at 0.125-0.25 μg/ml. In the Swedish Veterinary Antimicrobial Resistance Monitoring program (SVARM) a tiamulin cutoff for resistance in *B. hyodysenteriae* of >2 μg/ml has been used. This cutoff is also used at the National Veterinary Institute as a clinical breakpoint for resistance. A higher clinical tiamulin MIC breakpoint (> 4 μg/ml) was proposed by Rønne and Scanzer but has been questioned and in two publications a clinical breakpoint of >0.5 or >1 μg/ml for tiamulin tested by broth dilution has been discussed [22,23]. The arguments for lowering the clinical breakpoint are based on both pharmacokinetic data and clinical experience, although such information is limited. In this study the purpose of the wild type cutoff values are solely to monitor any change in the bacterial population, and not for use in interpreting the clinical outcome of treatment with a certain drug.

The suggested wild type cutoff values for monitoring of antimicrobial susceptibility in *Brachyspira hyodysenteriae* and the percentage of non-wild type isolates generated by these for the isolates included in this study are: tiamulin >0.25 μg/ml (8%), valnemulin >0.125 μg/ml (5%), tylosin >16 μg/ml (75%), tylvalosin >1 μg/ml (73%), lincomycin >1 μg/ml (74%) and doxycycline >0.5 μg/ml (13%). These percentages do not necessarily reflect clinical resistance. The wild type cutoff values are also shown as arrows in Figure 1. For *B. pilosicoli* there are few studies on antimicrobial resistance mechanisms and MIC distributions. To some extent the cutoff values proposed for *B. hyodysenteriae* may be applicable also for monitoring of *B. pilosicoli* but the MIC distributions differ slightly and the tylvalosin and lincomycin wild type MICs seem to be higher for *B. pilosicoli.*

## Conclusions

The antimicrobial susceptibility tests performed with the broth dilution method used in this study have over the years generated tightly grouped MIC populations for the field isolates and reproducible results for the control strain. This stable method is therefore well suited for monitoring of susceptibility in *Brachyspira* spp. To aid detection and monitoring of antimicrobial susceptibility wild type cutoff values that define the population of bacteria with no acquired phenotypically detectable resistance mechanism have been established for many “bug-drug” combinations [7]. In this study we have proposed wild type cutoff values for six antimicrobial agents for *B. hyodysenteriae* tested by broth dilution based on MIC distributions and the current knowledge on mechanisms of resistance in this species.

## Competing interests

The authors declare that they have no competing interests.

## Authors’ contributions

MP compiled the data and drafted the manuscript. AL and MP originally developed the broth dilution method and performed all susceptibility tests. HEU, BM and BB participated in the analysis of the results and helped to draft the manuscript. All authors read and approved the final manuscript.
